# Scalable multiplex co-fractionation/mass spectrometry platform for accelerated protein interactome discovery

**DOI:** 10.1038/s41467-022-31809-z

**Published:** 2022-07-13

**Authors:** Pierre C. Havugimana, Raghuveera Kumar Goel, Sadhna Phanse, Ahmed Youssef, Dzmitry Padhorny, Sergei Kotelnikov, Dima Kozakov, Andrew Emili

**Affiliations:** 1 Center for Network Systems Biology, Boston University, Boston, MA USA; 2 Department of Biochemistry, Boston University School of Medicine, Boston, MA USA; 3 Bioinformatics Program, Boston University, Boston, MA USA; 4 Laufer Center for Physical and Quantitative Biology, Stony Brook University, Stony Brook, NY USA; 5 Department of Applied Mathematics and Statistics, Stony Brook University, Stony Brook, NY USA

**Keywords:** Protein-protein interaction networks, Mass spectrometry, Isolation, separation and purification, Biochemical networks

## Abstract

Co-fractionation/mass spectrometry (CF/MS) enables the mapping of endogenous macromolecular networks on a proteome scale, but current methods are experimentally laborious, resource intensive and afford lesser quantitative accuracy. Here, we present a technically efficient, cost-effective and reproducible multiplex CF/MS (mCF/MS) platform for measuring and comparing, simultaneously, multi-protein assemblies across different experimental samples at a rate that is up to an order of magnitude faster than previous approaches. We apply mCF/MS to map the protein interaction landscape of non-transformed mammary epithelia versus breast cancer cells in parallel, revealing large-scale differences in protein-protein interactions and the relative abundance of associated macromolecules connected with cancer-related pathways and altered cellular processes. The integration of multiplexing capability within an optimized workflow renders mCF/MS as a powerful tool for systematically exploring physical interaction networks in a comparative manner.

## Abstract (web summary)

Co-fractionation/mass spectrometry (CF/MS) allows mapping protein interactomes but efficiency and quantitative accuracy are limited. Here, the authors develop a reproducible multiplexed CF/MS method and apply it to characterize interactome rewiring in breast cancer cells.

## Introduction

Proteins often physically associate to form higher order multimeric assemblies that perform key biochemical functions in different cell types and cell states^1,2^. Multiple experimental approaches have been devised to identify these biophysical interactions^3^, but most techniques (e.g., affinity purification, proximity labeling, or immunoprecipitation) involve selective protein tagging, which precludes the unbiased study of endogenous macromolecular networks. While isotopic labeling and the use of sophisticated computational scoring have emerged as potent means for enhancing the reliability of interactomic studies^4,5^, these strategies impose additional constraints that have limited their wider adoption. Thus, more efficient, and effective strategies for mapping protein interaction networks (PINs) by increasing assay throughput, automation, and quantitative accuracy remain desirable.

Biochemical fractionation-coupled to mass spectrometry (i.e., CF/MS) is a powerful alternative approach for the large-scale detection of native protein complexes in cellular extracts^6–11^. CF/MS involves the biophysical separation (e.g., chromatography) of endogenous macromolecules isolated from cell or tissue-derived soluble lysates, followed by liquid chromatography-tandem mass spectrometry (LC/MS) based identification of stably interacting proteins that co-elute together as components of intact multiprotein assemblies^1,12^. Since there is no exogenous introduction of genetic material or requirement of reagents for affinity purification, CF/MS can be used to examine macromolecular networks in a near physiological context starting from virtually any biological sample. Protein interaction network (PIN) coverage and accuracy are significantly improved by performing replicate CF/MS experiments and by inclusion of supporting functional association evidence^5–8,13^, but the former strategy is burdensome since it involves time-consuming, resource-intensive processing and LC/MS analysis of potentially hundreds of replicate fractions while the latter imposes bias^14^.

Here, we leverage isobaric tandem mass tag (TMT) sample barcoding together with automated processing of replicate biochemical fractionations as the basis of a multiplexed (mCF/MS) workflow that overcomes existing CF/MS drawbacks. We designed, optimized and validated a rigorous mCF/MS protocol that can markedly accelerate comparative interactome discovery by minimizing manual sample manipulation and total LC/MS instrument time while also eliminating the need for computational integration of functional annotation evidence for high confidence PIN scoring. We apply this enhanced workflow to decipher and compare the global protein interactomes of non-transformed mammary epithelia against the PIN of two breast cancer cell lines associated with triple-negative and luminal A molecular subtypes, revealing macromolecular complexes that appear to drive important malignant cell phenotypes. This breast cancer interactome resource is publicly available at https://www.bu.edu/dbin/cnsb/BrCa3CL/.

## Results

### Multiplex CF/MS Workflow

**Fig. 1:**
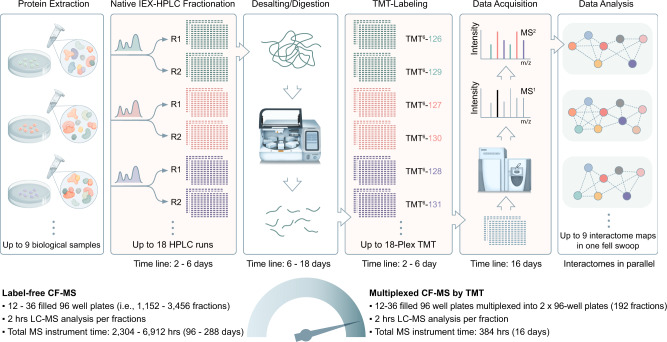
Multiplex CF/MS workflow. Schematic illustration of the main modular steps: 1 – native protein extraction; 2 - biochemical fractionation (replicates R1/R2); 3 - automated protein desalting and digestion; 4 - isobaric (TMT) labeling; 5 - LC/MS data acquisition; 6 – PPI/co-complex data analysis. For our use case, soluble protein extracts from cultured MCF10A, MCF7, and MDA-MB-231 cells were used to illustrate and benchmark the mCF/MS pipeline. The processing time for each modular step is shown in terms of total instrument usage as compared to a conventional label-free CF/MS procedure under otherwise identical conditions.

We designed and optimized a seamless mCF/MS workflow to multiplex the analyses of up to 18 independent biochemical fractionation experiments, implementing automated (robot-assisted) desalting and proteolytic digestion of protein samples coupled to isobaric (TMT) peptide labeling^15,16^ and pooling prior to standard LC/MS runs (Fig. 1). To achieve high-resolution separation of endogenous macromolecules, we first subjected soluble protein lysates extracted from cell (or tissue) biospecimens of interest (e.g., tumor-derived cell lines) to extensive multibed ion-exchange liquid chromatography (IEX-HPLC), as described previously^6,9^. Using an empirically optimized scheme, we applied a gentle salt gradient to preserve native macromolecule integrity while collecting up to 192 fractions (i.e., two 96-well plates) per biological sample. To ensure rigor (data reproducibility), we also performed multiple independent fractionations (duplicate IEX-HPLC runs per sample). Since CF/MS generates a substantial number of native protein fractions (e.g., potentially >1000 fractions in a replicate experiment comparing three cell lines), we implemented automated magnetic bead-based sample processing compatible with high peptide recovery and low reagent consumption (see Methods). We opted for paramagnetic carboxylic resin^17^, which is well-suited for reversed-phase protein desalting, followed by direct on-bead trypsin digestion in a 96-well plate format to preserve sample orthogonality. The use of a magnetic bead handling robot minimized sample consumption, time and labor prior to downstream manual sample processing steps. As outlined schematically in Fig. 1, the peptides generated from respective sample fractions are then individually barcoded using distinct TMT (e.g., 6-plex^16^) reagents and then pooled for LC/MS analysis, allowing both replicates and different samples to be studied in parallel to reveal both intra-replicate reproducibility and quantitative biological differences.

### Multiplex CF/MS enables multi-condition protein co-elution profiling

As a test use case, we performed a large-scale survey of PIN alterations in breast cancer by using mCF/MS to compare the composition and levels of protein macromolecules in three established human cell lines (mammary-tumor derived triple-negative MDA-MB-231, estrogen receptor-positive MCF7, and non-transformed MCF10A breast epithelial cells). The cell lines were grown in near-identical tissue culture conditions and subjected to extensive IEX-HPLC fractionation in two replicates. After automated protein digestion, TMT-6-plex labeling and pooling of multiplexed samples, we performed quantitative LC/MS analyses to examine the nature and extent of macromolecular rewiring that occurs in the transformed cell state.

**Fig. 2:**
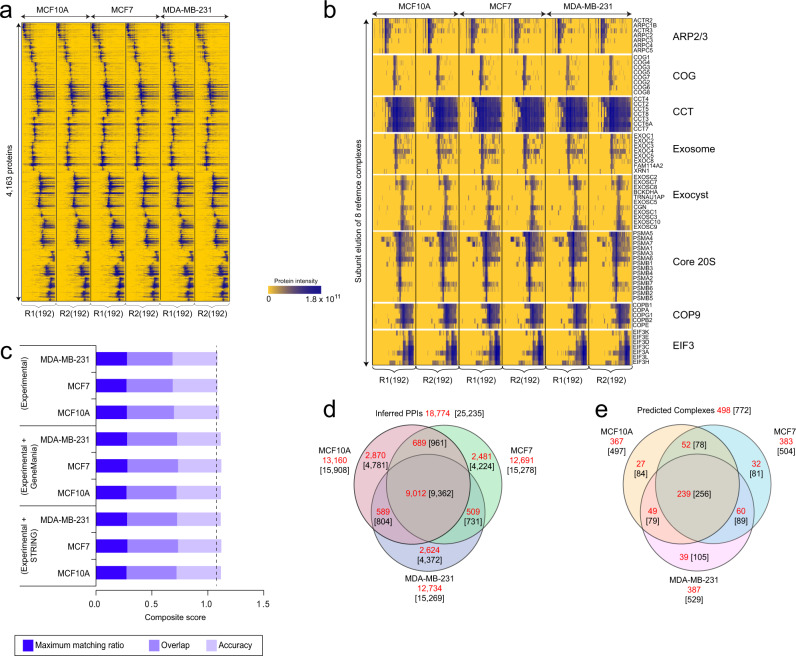
Comparison of protein interactomes from three mammary cell line models of breast cancer. **a** Hierarchical clustering of replicate (R1 & R2) IEX-HPLC profiles (192 protein fractions) obtained for MCF10A, MCF7 and MDA-MB-231 cells profiled by mCF/MS. Blue shading indicates protein ion intensities recorded by LC/MS. **b** Co-elution profiles of annotated subunits of representative reference complexes. **c** High EPIC Composite Scores (summed maximum matching ratio, overlap, and accuracy metrics) of complexes corresponding to the three cell lines surveyed based on the experimental (mCF/MS) data alone (unbiased), which is only marginally boosted after integration of external functional evidence (from STRING or GeneMANIA). Source data are provided as a source data file. **d** Venn diagrams depicting the distribution of inferred high-confidence PPIs, both unique and shared among the three cell lines, including total (*black*) and previously reported associations (*red*). **e** Venn diagram depicting the distribution of protein complexes derived from partitioning the breast cancer PINs (*black*), including those matching annotated assemblies (*red*) curated by the CORUM, IntAct, GO, or Reactome databases.

In total, the coelution profiles of 4613 soluble proteins were identified and quantified with high confidence (Supplementary Data 1), with the vast majority (4599, 99.7%) detected across all six samples without missing values (Fig. 2a). Notably, sample multiplexing significantly reduced (in this case, by 6-fold) the total number of LC/MS injections, greatly accelerating data acquisition while using less instrument time (e.g., 2 weeks as opposed to ~14–18 weeks using our standard label-free method). Our mCF/MS workflow also consumed substantially less starting material (total protein extract) while still exhibiting high peptide reporter ion intensity, signal-to-noise and protein co-elution profile reproducibility (average Pearson correlation ≥0.95 between replicates; Supplementary Fig. 1a, b). For example, we observed consistently high pairwise co-elution profile correlations among known (annotated) complex subunits as compared to randomized protein pairs (Supplementary Fig. 1c). Notably, the annotated components of many representative multi-protein assemblies (e.g., ARP2/3, 20 S proteasome, CCT/TRiC, COG, Exocyst, COP9, Exosome, EIF3) were found to reproducibly coelute (Fig. 2b), attesting to the overall reliability of the entire mCF/MS workflow.

### Mapping high resolution differential protein interaction networks in breast cancer cells

To rigorously infer high-confidence PIN from these global co-fractionation data, we applied a stringent supervised classifier scoring model, implemented within our extensively benchmarked EPIC software^13^, to assess co-elution profile similarity and predict PPIs relative to a large set of reference “gold standard” protein complexes curated by Gene Ontology (GO)^18^, IntAct^19^, and CORUM^20^. To avoid any potential bias, we did not use or integrate any functional evidence in our scoring scheme. Rather, we evaluated the overall performance of the mCF/MS data to reliably infer protein complex memberships by calculating a summary Composite Score comprised of three independent evaluation metrics commonly used to assess the accuracy of macromolecule predictions relative to annotated complexes^8,13,21^, i.e., (i) Maximum Matching Ratio, (ii) Overlap and (iii) Prediction Accuracy (Fig. 2c).

This rigorous benchmarking confirmed that our mCF/MS procedure generated reliable PIN data for each of the three cell lines. Notably, a third (9327) of the 25,235 PPIs detected by EPIC (Supplementary Data 2) were preferentially detected in breast cancer (MCF7 and MDA-MB-231) cells (Fig. 2d), suggesting potential involvement in the establishment of the oncogenic phenotypes. Moreover, most (73%, or 18,774) of the PPIs detected by joint mCF/MS analysis of the three cell lines had at least one supporting reference in the literature or a public repository (Supplementary Fig. 2a). Using an empirically optimized EPIC cutoff (0.625 score threshold), mCF/MS-derived macromolecules attained >80% precision (FDR = 0.2) against reference co-complexes curated in the CORUM database (Supplementary Fig. 2b), pointing to the overall reliability of mCF/MS for global PIN mapping. Strikingly, whereas supporting functional evidence from external annotation repositories is often required to boost prediction reliability using standard co-fractionation workflows^6–8,11,13^, our mCF/MS experimental data alone produced comparably high Composite Scores that were not significantly enhanced by the additional inclusion of associating evidence from STRING^22^ or GeneMANIA^23^ (Fig. 2c). Out of the 772 putative multi-protein assemblies identified from our mCF/MS data (Fig. 2e, *black*; Supplementary Fig. 3; Supplementary Data 3), most (498, 64.5%) showed significant overlap (Simpson’s similarity index ≥0.45) to a previously reported (annotated) protein complex (Fig. 2e, *red*). The remaining set of 274 novel multi-protein assemblies (Supplementary Fig. 3) illustrate the potential of the mCF/MS platform for biological discovery.

**Fig. 3:**
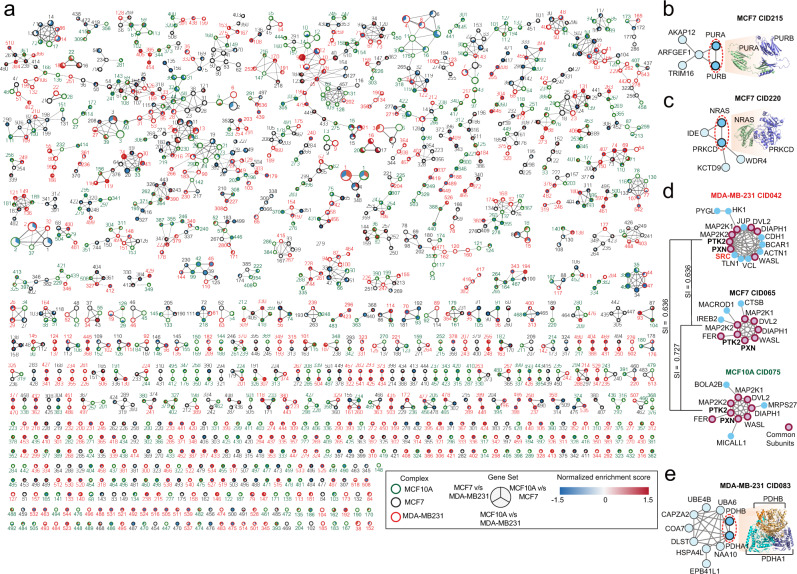
Protein interaction network map and exemplar protein complexes identified in breast cancer cells. **a** A global protein interaction network map illustrating enrichment of macromolecular assemblies (related to Supplementary Fig. 3 and Supplementary Table 3) and based on abundance (normalized TMT reporter ion intensities, red: high and blue: low) in each of the 3 cell lines. Structural models depicting binary component interaction interfaces of members of select complexes detected in MCF7 cells, **b** CID.215 and **c** CID.220. **d** An exemplar complex depicting rewired interactions (edge) between Src, PTK2, and PXN among other members (nodes) across the 3 cell lines. Src is recruited to PTK2 and PXN preferentially in the MDA-MB-231 cells with implications in breast tumor plasticity. Red nodes indicate common/shared subunits. Simpson’s similarity index depicts the degree of protein complex similarity between the 3 cell lines. **e** Structural model depicting binary component interaction interfaces of members of an exemplar complex, CID.083, detected in MDA-MB-231 cells.

The precise TMT-labeling revealed quantitative changes in the levels (relative abundance) of multiple protein complexes between the control MCF10A and cancerous MCF7 and MDA-MB-231 cell lines (Fig. 3a). We observed a widely distributed protein complex similarity index (Simpson’s Index; SI) profile among the three cell lines (Supplementary Fig. 4a–c), revealing several highly conserved complexes including the well characterized anaphase promoting complex or cyclosome (APC/C)^24,25^ and human PAF1 complex (hPAF1c)^26,27^ (SI = 0.8–1.0, Supplementary Fig. 4d, e), as well as a considerable number of differential macromolecular assemblies (SI < 0.45) relevant to breast cancer cells. Some of the exemplar differential complexes relevant to oncological contexts include CID.105 (PAPSS2, STXBP2, GGCT, PLOD2, CTSB, COLGALT1, and ZNHIT3) enriched in MDA-MB-231 cells (Supplementary Fig. 4f) and CID.215 (AKAP12, ARFGEF1, TRIM16, PURA, PURB), CID.220 (NRAS, IDE, PRKCD, KCTD9, WDR4) and CID.161 (UBA52, SERPINH1, PPA2, PCBP1, PLEC, and CTSD) that were preferentially found in the ER + MCF7 cells (Fig. 3b–e; Supplementary Fig. 4g). PURA and PURB are nucleic acid-binding proteins that form nucleoprotein complexes associated with hematologic malignancies and hyperproliferation^28,29^. Structural modeling revealed a potential interaction interface situated in the N-terminal regions of both polypeptide subunits (Fig. 3b), indicative of a heterocomplex potentially formed in conjunction with RNA/DNA. AKAP12, another member of this same complex, plays a significant role in phosphorylation-dependent cell cycle progression and nucleocytoplasmic shuttling to facilitate DNA repair^30,31^, implying alteration of these activities in ER + breast cancer. Likewise, while PRKCD is known to regulate RAS signaling extensively in several tumor types including breast cancer^32^, our mCF/MS results suggest an unexplored physical role in modulating downstream MAPK signaling^33^ (Fig. 3c).

Within the set of differential protein assemblies exhibiting rewiring in the breast adenocarcinoma-derived MDA-MB-231 cells, we enriched a well-characterized multiprotein assembly involving Src, PTK2 (FAK) and PXN, corroborating previously reported molecular mechanisms underlying breast tumor plasticity^34,35^ (Fig. 3d). Among other complexes altered in MDA-MB-231, we noted CID.083 (PDHB, PDHA1, NAA10, HSPA4L, EPB1L1, DLST, COA7, CAPZA2, UBE4B, UBA6) (Fig. 3e) whose members include a heterotetrameric pyruvate dehydrogenase subcomplex (PDHA1, PDHB), mitochondrial matrix components (e.g., CAO7, DLST, HSP4L) and non-resident mitochondrial factors (e.g., UBE4B, UBA6). The association of the ubiquitin-like modifier enzyme UBA6 aligns with recent studies implicating UBA6-specific substates in mitochondrial dysfunction^36^ and the enhanced metabolic characteristics of triple-negative breast cancers^37,38^, and may be attributed to differential regulatory phosphorylation by pyruvate dehydrogenase kinases, PDHKs and phosphatases, PDP1/2^39^.

To gain broader functional insights into the cellular pathways and processes impacted by the differential protein assemblies detected by mCF/MS, we performed systematic functional enrichment analysis. Significant (adj. *p* < 0.05) alterations were seen in the relative abundance of dozens of multi-protein assemblies linked to 98 different cellular pathways and processes (Supplementary Data 4), which were broadly annotated into 64 major functional themes (Supplementary Fig. 5). As anticipated, a number of established oncogenic signaling pathways linked to cell survival and invasiveness were enriched in the breast cancer cells. These included assemblies preferentially detected in MDA-MB-231 cells with components implicated in Hedgehog and ERBB signaling as previously reported for triple negative cancers^40–42^, and complexes enriched for WNT and FGFR2 signaling components in both MCF7 and MDA-MB-231 cells. Additionally, multi-protein assemblies with components involved in RNA processing/mRNA splicing, angiogenesis and central metabolism (e.g., oxidative phosphorylation and carbohydrate utilization) were enriched in both cancer lines (Supplementary Data 4), implicating these in mediating oncogenic phenotypes.

Since protein interaction networks and, consequently, cellular and physiological phenotypes are often profoundly impacted by genomic aberrations associated with tumor progression^43,44^, we cross-referenced the components of our mCF/MS-derived macromolecular networks against the Cancer Genome Atlas (TCGA)^45^ to link cancer-causing genomic alterations to the differential assemblies we detected in the cancer cell lines. This led us to identify molecular alterations present in triple-negative (126 cases) and ER-positive (622 cases) breast cancer, including missense and nonsense mutations, genomic insertions, and deletions that map specifically onto the PIN and multi-protein assemblies we detected in MCF-7 and MDA-MB-231 (Supplementary Figs. 6, 7). Overall, clinical genomic aberrations were associated with 2263 PPIs in MDA-MB-231, and 2038 PPI in MCF7. In addition to the extensive TP53 mutations cataloged in both ER-positive and triple negative cases, multiple genes (e.g., CDH1, AKT1, NCOR1, DYNC2H1, BIRC6, MYO18B) encoding the interacting components of cancer cell line-specific PPIs displayed significant rates of mutation in each the respective breast cancer molecular subtypes (Supplementary Figs. 6, 7), suggesting that this mutational burden impacts multi-protein complexes directly and consequently elicits distinct malignant phenotypes as noted recently by ref. ^46^.

## Discussion

The automated processing and isobaric barcoding of biochemical fractions implemented in the mCF/MS workflow represents a substantive advance in speed, efficiency and efficacy for comparative exploration of cellular interactomes relative to standard label-free^6–9,11,13^ or alternate SILAC-based CF/MS methods^4,5^. Our multiplexing strategy shows good performance in benchmarking tests even after sample downscaling (less resource consumption), conferring additional advantages over existing workflows^4–12^. In comparison, our previous studies of alternate human embryonic and cancer cell lines (HEK293, HeLa) implementing orthogonal 2-dimensional chromatography workflows that generated >1000 biochemical fractions^6^ detected only half as many (~14,000) PPIs, of which just a small subset was deemed to be cancer cell line-specific, underscoring the high overall utility of mCF/MS relative to standard approaches.

Cellular PIN rewiring is governed by genomic alterations and dynamic changes in protein expression, post-translational modification, and subcellular localization^47–51^. Robust detection and quantification of co-eluting proteins across different samples is essential to optimally infer differential protein complexes. Capturing protein expression information is therefore paramount to protein complex discovery. A poorly expressed protein in one cell line for instance, may potentially go undetected due to the stochastic nature of MS-based data acquisition leading to a presumably artefactual omission of the protein from a specific protein complex. Our mCF/MS workflow here is technically advantageous in boosting and enabling the reproducible detection of proteins at otherwise near- or below-noise threshold. However, it is important to note that the relative expression of proteins between cell lines, e.g., cancer and non-cancer lines, may account for potential intrinsic mechanisms deployed to either favor or curb molecular association and elicit specific cellular functions. Such differences in protein abundance are especially well documented in cancer cells and tissue including breast cancer^52^. We, therefore, reasoned that the quantitative capabilities of mCF/MS would enable insights into differential PPI formation among oncoproteins and the accumulation of key macromolecules in breast cancer cells, missed by standard qualitative CF/MS workflows (Fig. 3a). In principle, using new generation 16-18-plex TMT reagents^15,53^, mCF/MS experiments encompassing replicate analyses of up to 8 to 9 distinct biospecimens can be accomplished in a similar timeframe. This highlights the unique advantage of barcoding and automation for improving throughput and facilitating quantitative comparisons of larger sample cohorts^15,53^. We conclude that mCF/MS should empower mapping of the basic macromolecular machinery of tumors and other cell types.

## Methods

### Cell culture and preparation of native cellular protein extracts

Human mammary tissue-derived cell lines were procured from American Tissue Culture Collection (ATCC, VA, USA). MCF10A cells (cat. # CRL-10317, ATCC) were cultured in Dulbecco’s modified Eagle’s medium (DMEM)-F12 medium supplemented with 0.5 mg/mL hydrocortisone, 100 ng/mL cholera toxin, 20 ng/mL EGF, 10 μg/mL insulin, and 5% horse serum (Fisher Scientific). MCF7 (cat. # HTB-22, ATCC) and MDA-MB-231 (cat. # HTB-26, ATCC) cells were cultured in high-glucose DMEM supplemented with 10% (v/v) fetal bovine serum (Fisher Scientific) and 10 μg/mL insulin (MCF7 only). All cells were cultured in 5% CO_2_ at 37 °C. Replicate cell cultures were harvested at sub-confluence by scraping in 1x phosphate buffered saline. Cells were centrifuged at 7000 × *g* for 1 min, snap-frozen in liquid nitrogen and stored at −80 ^o^C until use. Cell lysis was performed by resuspension and Dounce homogenization in buffer containing 10 mM Tris-HCl, 250 mM sucrose, 5 mM MgCl_2_, 1 mM dithiothreitol (DTT), 0.1% (v/v) dodecyl-β-D-maltopyranoside (DDM) and 1× Complete Protease and Phosphatase Inhibitor Cocktail (Roche). The homogenates were treated with Turbonuclease (100 units/mL) (Accelagen) for 30 min at 4 ^o^C, clarified by centrifugation at 18,000 × *g* for 20 min at 4 °C, quantified by Bradford assay (Bio-Rad) and adjusted to 6.0 mg protein/mL prior to fractionation.

### Biochemical fractionation

To enhance resolution and streamline downstream sample processing, we scaled down our previously optimized semi-preparative triple phase IEX-HPLC methodology^6^. Specifically, protein extracts from each cell line replicate were fractionated independently by triple-phase ion-exchange chromatography using an Agilent 1260 Infinity binary HPLC system consisting of a stacked assembly in which a weak anion exchange PolyWAX LP column (200 × 2.1 mm i.d., 5 µm, 1000-A; PolyLC Inc., MD, USA) was connected in tandem to two weak cation exchange PolyCAT A columns (each 200 × 2.1 mm i.d., 5 µm, 1000-A; PolyLC). The columns were conditioned in buffer A (10 mM Tris-HCl, pH 7.6, 3 mM NaN_3_, 1% (v/v) glycerol) prior to loading 1.5 mg of protein extract per replicate. For each replicate, 192 80-µL fractions were collected (two 96-well plates) at a flow rate of 0.125 mL/min using a gradient elution of 0–67% buffer B (buffer A + 1.5 M NaCl) from 8–80 min, followed by 67–100% between 80–96 min. Protein elution was monitored by determining the UV absorbance at 280 nm. The collection of low sample volume allowed direct protein denaturation in a manageable final volume compatible with the downstream automated desalting and digestion on an automated Kingfisher magnetic purification instrument (Thermo Fisher Scientific).

### Automated desalting and digestion of co-fractionated proteins

The IEX-HPLC co-fractionated samples were desalted and digested with trypsin in 96-well format using the KingFisher Apex instrument (Thermo Fisher Scientific). Protein fractions (≤10 µg total proteins) were denatured using 4 M urea, reduced with 20 mM DTT for 30 min and alkylated with 20 mM iodoacetamide for another 30 min in the dark at room temperature. After the reaction was quenched with 10 mM DTT for 15 min at room temperature, the reduced and alkylated protein fractions were desalted using 100 µg of an equal mixture of hydrophobic and hydrophilic SeraMag SpeedBead carboxylate-modified magnetic particles (GE Life Sciences), followed by on-bead digestion using sequencing-grade trypsin (Pierce) in a 100 mM triethylammonium bicarbonate solution for 8 h at 37 ^o^C. After drying in SpeedVac, samples in the plates were labeled using a unique TMT-6plex reagent (ThermoFisher Scientific) according to the manufacturer’s instructions with slight modification to minimize TMT reagent consumption. Briefly, a total of 5 mg of reagent per channel was used to equally label the 192 ion-exchange protein fraction digests (i.e., 25 µg total reagent per 5 µg protein digests in each well), which were then pooled (totaling 192 multiplex samples) and dried by SpeedVac for subsequent analysis by LC-MS/MS.

### Nanoflow LC-MS/MS data acquisition and analysis

The TMT-labeled peptides were solubilized in mobile phase A (2% acetonitrile, 0.1% formic acid), loaded using an EasyNanoLC 1200 HPLC pump onto a C18 trap column (75 µm i.d × 2 cm, Acclaim PepMap100, Thermo Fisher Scientific), and resolved on an EASY-spray column (75 µm i.d × 50 cm, PepMap RSLC C18, Thermo Fisher Scientific) using a 90-min gradient (7–35% over 60 min, 35–60% over 30 min) of mobile phase B (80% acetonitrile, 0.1% formic acid) at a 250 nL/min flow rate prior to injection into the Q Exactive Orbitrap HF mass spectrometer (Thermo Fisher Scientific). The instrument was operated in positive ion mode using an electrospray voltage of 2100 V. High-energy collision dissociation (HCD) fragmentation data were acquired in data-dependent acquisition (DDA) mode. Precursor ions (MS^1^, 300–1500 m/z) were scanned at a resolution of 120,000 at *m/z* 200, using an injection time of 60 ms with an AGC target of 3 × 10^6^ ions. The top 10 precursor ions were selected for fragmentation (MS^2^) and scanned at a resolution of 30,000 at *m/z* 200, using an injection time of 60 ms and an AGC target of 1 × 10^5^ ions.

The raw data were processed using MaxQuant (version 1.6.1.0). All spectra were searched using the Andromeda search engine against a FASTA file of the *Homo sapiens* proteome (dated January 2021; 20,294 entries) downloaded from UniprotKB. Oxidation and acetylation were specified as variable modifications, while carbamidomethylation was specified as a fixed modification. For quantification at tandem MS level, reporter ion MS^2^ with pre-defined 6-plex TMT labels and reporter mass tolerance of 0.003 Da were set as relevant parameters. Trypsin/P was specified as the proteolytic enzyme, with up to two missed cleavage sites allowed. The precursor and fragment ions tolerance were set to 4.5 and 20 ppm, respectively. Match between runs was enabled, with all other MaxQuant settings set to default. Batch-specific correction factors for TMT isotope ratios were entered to correct for variable channel intensities. Protein and peptide identification confidence threshold were set to an FDR of 1%.

### Computational scoring of PPIs and protein complexes

The search files, containing batch-corrected peptide MS^2^ reporter ion intensities corresponding to all fractions, were processed in EPIC^13^ to predict PPIs and protein complexes, using a random forest classifier with default parameters, as described previously^8,13^. Briefly, after proteins detected in only a single fraction were discarded and the resulting matrix was subjected to column-wise and row-wise normalizations to mitigate fraction-bias^8,13^. Annotated protein complexes from Gene Ontology^18^, IntAct^19^, and CORUM^20^ were used for training and evaluation. Co-elution scores were calculated for each cell line using five correlation metrics (Euclidean, Bayes, Jaccard, Apex, Mutual information)^8,13^. Predictions based on mCF/MS data alone gave the best Composite Score results at an EPIC score cut-off of 0.625 and were compared to those obtained with functional associations (excluding physical interactions to avoid circularity) collected from STRING^22^ and GeneMANIA^23^. Protein complex memberships were defined using ClusterOne^21^. Simpson’s similarity index was used to estimate the overlap of predicted complexes between cell lines, then among annotated complexes from the literature and publicly available repositories. Pearson’s correlation coefficients (R^2^) were calculated to determine reproducibility across intra-replicate IEX-HPLC experiments.

### Mapping PPIs to public interactome repositories, literature and TCGA database

To evaluate the extent to which our predicted PPIs recapitulate previously published/annotated interactions, we mapped our binary protein interactions to human protein interactions from various public repositories and literature. Physical and functional protein associations were downloaded from curated public repositories including STRING v11.0^22^, GeneMania^23^, HumanNet v.2.0^54^, BioGrid (July 2021)^55^, IrefIndex v.11^56^, CORUM^20^, Reactome^57^, Gene Ontology^18^, IntAct^19^, hu.MAP2.0 PPIs^58^ (confidence cut off of 0.02), BioPlex 3.0^59^, consensus human CF/MS interactome^14^, and high-throughput co-complex pairwise protein interactions from CF/MS^6,7^ were used for cross-validations. UniprotKB accession ID were used as a common identifer.

The R package ‘Maftools’^60^ was used to map TCGA^45^-cataloged mutations onto PPI genes associated with the MDA-MB-231 and MCF7 breast cancer cell lines. Mutations in 16,803 genes across 126 triple-negative and 622 ER-positive breast cancer samples were cross-referenced to infer the frequency of mutations as well as the mutational variants (SNPs, deletions and insertions) linked specifically to the PPI genes.

### Structural modeling of protein interactions

The structural models of protein complexes were prepared using a combination of AlphaFold2^61^ and ClusProTBM^62,63^. For AlphaFold2, the multiple sequence alignments were prepared using the MMseqs2 tool^64^ (Version 13-45111), and model selection was done based on the cutoff value of 10.0 for the Predicted Aligned Error (PAE) of interface residues. For ClusProTBM, the homology search was performed on a PDB100 database using hhsearch and the models were selected using probability cutoff of 99% and coverage cutoff of 75%. PDB templates for the computational structural models shown in Fig. 3 and Supplementary Fig. 4 are 2V55 (ROCK-1:RhoE co-complex)^65^, 1NI4 (Pyruvate dehydrogenase)^66^ and 2X0B (Angiotensinogen:Renin co-complex)^67^.

### Gene set enrichment analyses

A comprehensive compiled list of Human Pathway annotations maintained by Bader Lab containing 4457 genesets (Ver. January 01, 2022) was downloaded from http://download.baderlab.org/EM_Genesets/January_01_2022. Geneset enrichment analysis (GSEA)^68^ of predicted complexes from each cell line was performed using the normalized average protein intensity profiles. Genesets were restricted to 3925 pathways annotated with 3–500 proteins. Complexes enriched for pathways (*p* < 0.05) were visualized in Cytoscape^69^ using the Enrichment map^70^ and Auto Annotate^71^ plugins.

### Reporting summary

Further information on research design is available in the Nature Research Reporting Summary linked to this article.

## Supplementary information

Supplementary Information

Description of Additional Supplementary Files

Supplementary Dataset 1

Supplementary Dataset 2

Supplementary Dataset 3

Supplementary Dataset 4

Reporting Summary

## Data Availability

The mass spectrometry proteomics data (including both raw MS data and processed MaxQuant output) have been deposited to the ProteomeXchange Consortium^72^ via the PRIDE^73^ partner repository with the dataset identifier PXD027704. The breast cancer interactome resource is available at https://www.bu.edu/dbin/cnsb/BrCa3CL/. Computational structural models in Fig. 3 and Supplementary Fig. 4 were generated using the following PDB entries as template; 2V55 (ROCK-1:RhoE co-complex)^65^, 1NI4 (Pyruvate dehydrogenase)^66^ and 2X0B (Angiotensinogen:Renin co-complex)^67^. Source data are provided with this paper.

## Source data

Source Data
