# Supplementary material for: Scalable multiplex co-fractionation/mass spectrometry platform for accelerated protein interactome discovery
Source: Nat Commun. 2022 Jul 13;13:4043. doi: 10.1038/s41467-022-31809-z (PMC9279285; doi:10.1038/s41467-022-31809-z)
Supplement: Supplementary file 1 — Supplementary Information [file 41467_2022_31809_MOESM1_ESM.pdf]

Supplementary Information: Scalable multiplex co-fractionation/mass spectrometry platform for accelerated protein interactome discovery

Havugimana et al.

a

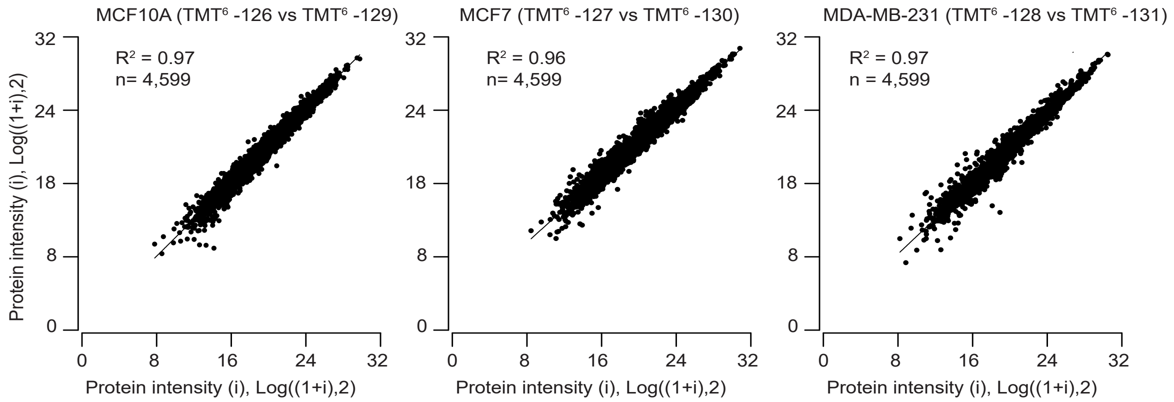

b

Zero-Order Correlation Coeffients between Human Breast-derived Cell Lines Proteomes

		1	2	3	4	5	6
**1**	MCF10A replicate1 (n=4,612)	---					
**2**	MCF10A replicate2 (n=4,612)	**0.97**	---				
**3**	MCF7 replicate1 (n=4,613)	0.85	0.84	---			
**4**	MCF7 replicate2 (n=4,610)	0.84	0.8	**0.98**	---		
**5**	MDA-MB231 replicate1 (n=4,608)	0.93	0.92	0.87	0.83	---	
**6**	MDA-MB231 replicate2 (n=4,608)	0.93	0.91	0.87	0.84	**0.98**	---

Note: Bold values indicate intra-replicate reproducibility, n = number of proteins detected per run.
Correlation analysis was performed using Built-in-functions of Microsoft Office Excel 2016.

c

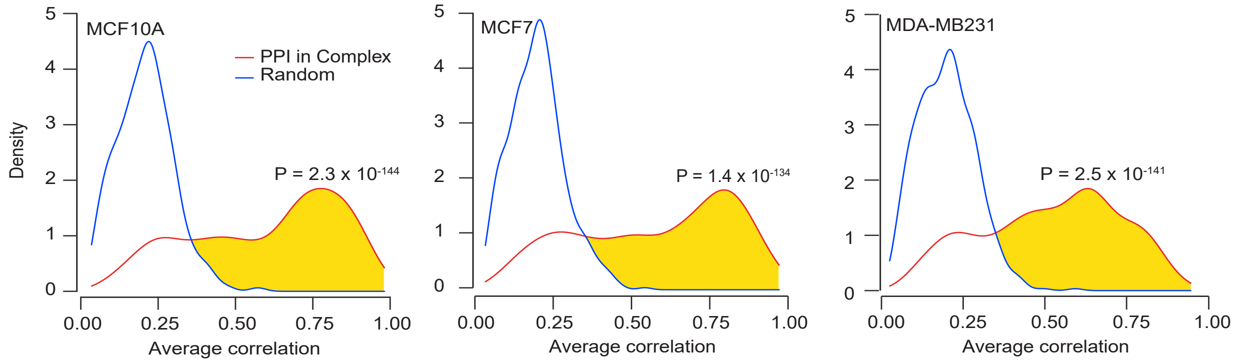

Supplementary Figure 1: a) Correlation plots showing Pearson's correlation coefficients calculated for replicate protein TMT reporter ion intensities in each cell line. **b**) Complete set of Pearson's coefficients based on TMT reporter ion intensities, including cross-cell line replicate comparisons. **c)** Average pairwise correlation (two-sided student's t-test; not adjusted for multiple comparisons) of protein pairs derived from annotated complexes of CORUM against randomized protein pairs for the indicated cell lines. High correlation for a substantial number of protein pairs belonging to annotated complexes alone (yellow) is evident. Source data are provided as a source data file.

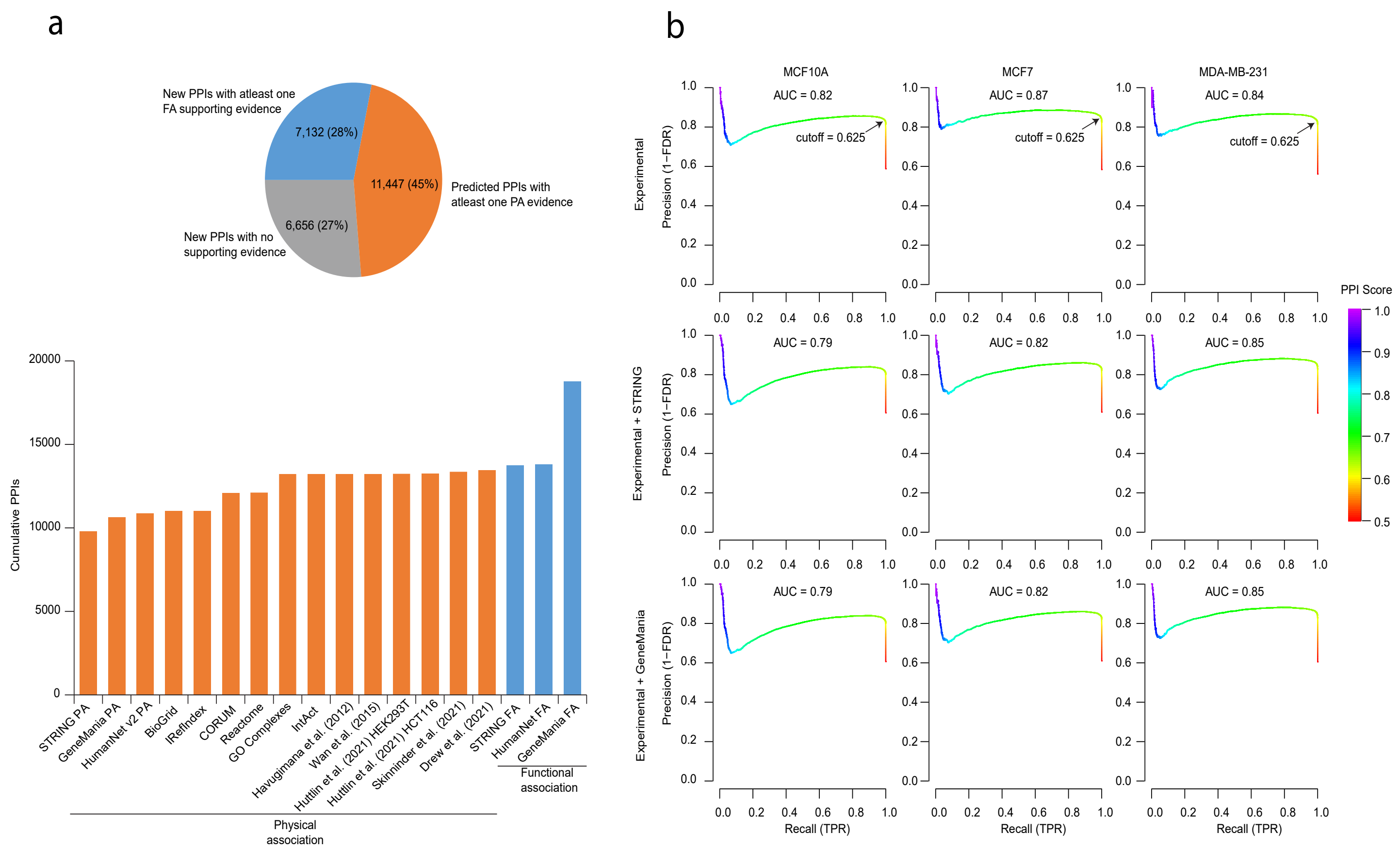

Supplementary Figure 2: a) Proportion of PPIs with supporting annotated evidence on physical associations (PA; orange bar/pie) and functional association (FA; blue bar/pie) as reported in different curated public databases and the published literature[1-5]. **b)** Precision-Recall plots for EPIC-predicted PPIs benchmarked against reference complexes derived from CORUM. Precision-Recall curves are plotted individually for PPIs inferred for each cell line at an EPIC score cut-off 0.625 with or without the integration of functional evidence from GeneMania and STRING. Source data are provided as a source data file.

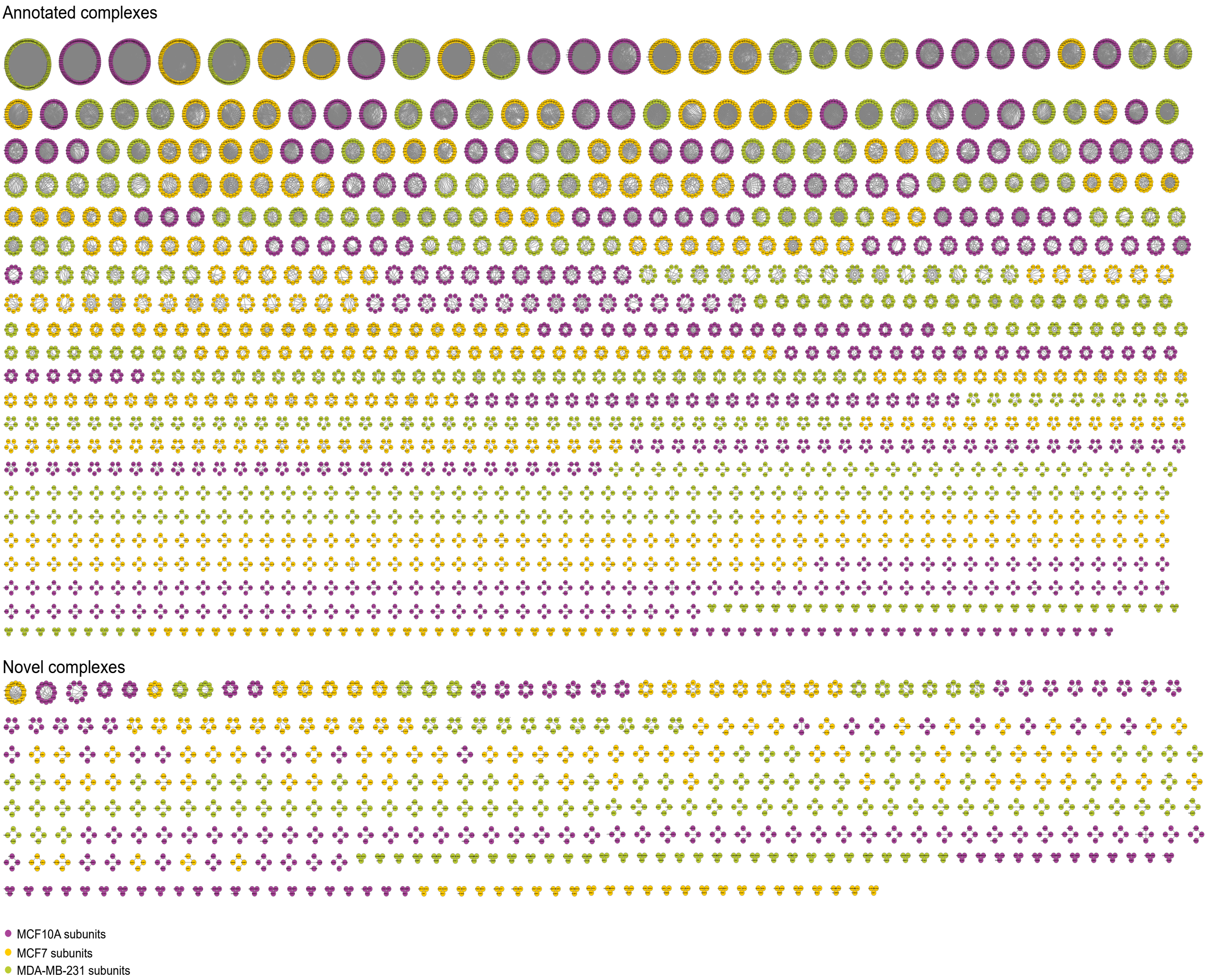

Supplementary Figure 3: Landscape of protein interaction networks identified from all three cell lines by mCF/MS. Yellow, green, and purple nodes represent protein subnetworks found by mCF/MS analyses in MCF7, MDA-MB-231, and MCF10A cells, respectively.

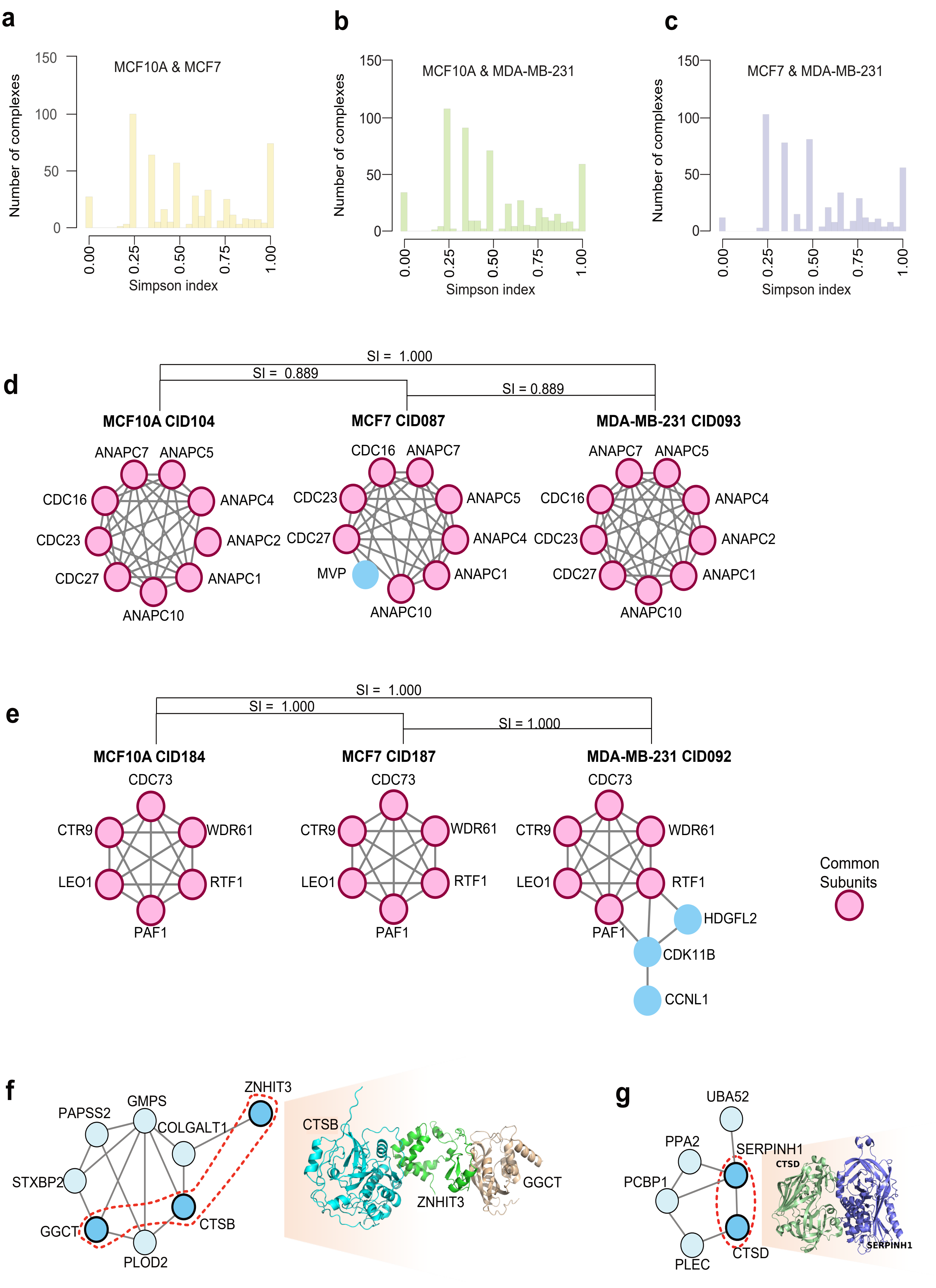
a
MCF10A & MCF7
Number of complexes
Simpson index
b
MCF10A & MDA-MB-231
Number of complexes
Simpson index
c
MCF7 & MDA-MB-231
Number of complexes
Simpson index
d
SI = 1.000
SI = 0.889
SI = 0.889
MCF10A CID104
MCF7 CID087
MDA-MB-231 CID093
ANAPC7 ANAPC5 CDC16 ANAPC4 CDC23 ANAPC2 CDC27 ANAPC1 ANAPC10
CDC16 ANAPC7 CDC23 ANAPC5 CDC27 ANAPC4 MVP ANAPC1 ANAPC10
ANAPC7 ANAPC5 CDC16 ANAPC4 CDC23 ANAPC2 CDC27 ANAPC1 ANAPC10
e
SI = 1.000
SI = 1.000
SI = 1.000
MCF10A CID184
MCF7 CID187
MDA-MB-231 CID092
CDC73 CTR9 WDR61 LEO1 RTF1 PAF1
CDC73 CTR9 WDR61 LEO1 RTF1 PAF1
CDC73 CTR9 WDR61 LEO1 RTF1 PAF1 HDGFL2 CDK11B CCNL1
Common Subunits
f
ZNHIT3 GMPS PAPSS2 COLGALT1 STXBP2 GGCT CTSB PLOD2
CTSB ZNHIT3 GGCT
g
UBA52 PPA2 SERPINH1 PCBP1 CTSD PLEC
CTSD SERPINH1

Supplementary Figure 4: Simpson's similarity index (SI) depicting global protein complex similarity between **a)** MCF10A vs MCF7, **b)** MCF10A vs MDA-MB-231 and **c)** MCF7 vs MDA-MB-231. Source data are provided as a source data file. Exemplar complexes namely, **d)** APC/C and **e)** hPAF1c, found conserved in all three cell lines. Red nodes indicate common/shared subunits. Exemplar protein complexes for **f)** MDA-MB-231 (CID.105) and **g)** MCF7 (CID.161) showing the interactions (edges) between member proteins (nodes). Structural models depict the interaction interfaces between the indicated proteins (dashed red line).

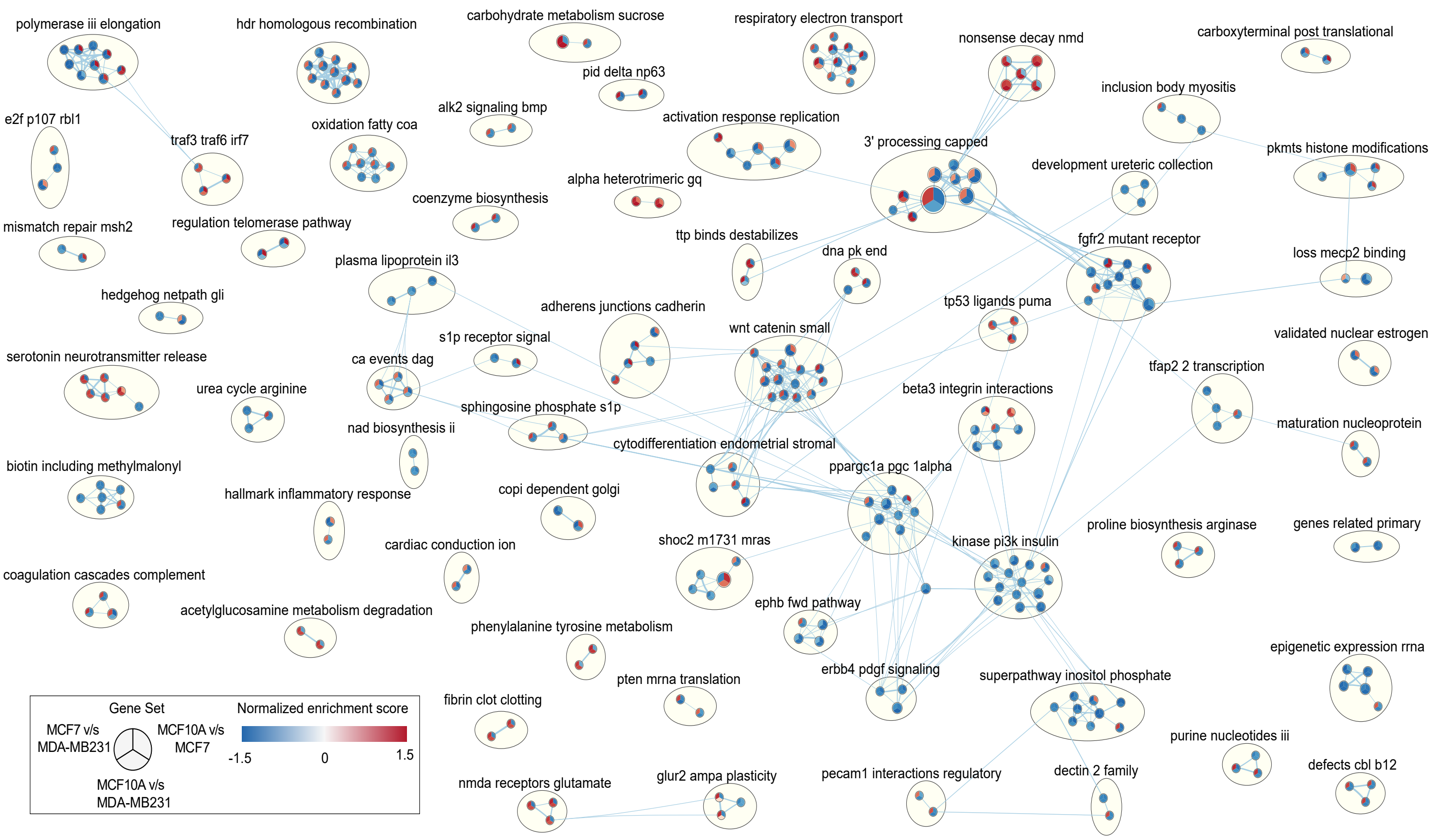

Supplementary Figure 5: Functional Geneset Enrichment Analyses (GSEA) depicting a network map of pathways and processes (nodes) enriched by members of protein complexes from the non-transformed mammary epithelial line, MCF10A, triple-negative breast cancer MDA-MB-231 and ER-positive breast cancer MCF7 lines. Red indicates positive enrichment while blue denotes negative enrichment for the respective cell lines. Genes/proteins shared between the enriched pathways are denoted by edges. Groups of nodes representing several related pathways were assigned common biological themes and were curated manually.

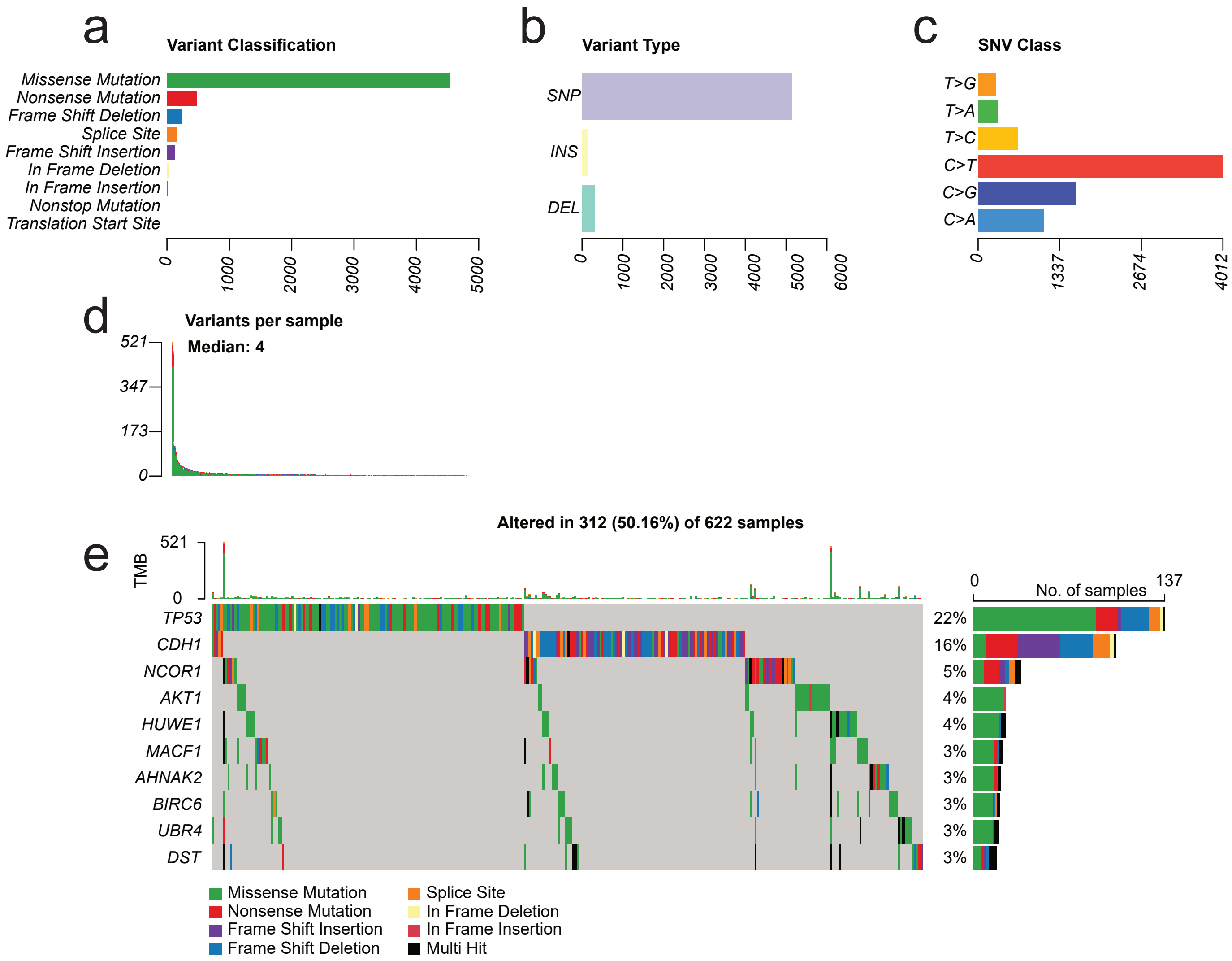

Oncoplot showing the top10 most frequently mutated genes inTCGA ER+ samples for genes in the MCF7 exclusive PPI

Supplementary Figure 6: The mutational landscape of ER-positive breast cancer PPIs as derived from the TCGA-database, showing in **a)** and **b)** the quantitative distribution of the specific variant types and broader classes among 312 out of 622 clinical ER-positive breast tumor cases. **c)** The type of single nucleotide variant (SNV) and associated frequencies are indicated. **d)** Bar-plot indicating the number of variants (median =4) sequenced for each tumor sample and includes the specific variant types (color-code same as in **a.**). **e)** Oncoplot showing the top 10 MCF7-associated PPI genes most frequently mutated in ER-positive breast cancer cases along with the various types of mutations as catalogued in TCGA. Source data are provided as a source data file

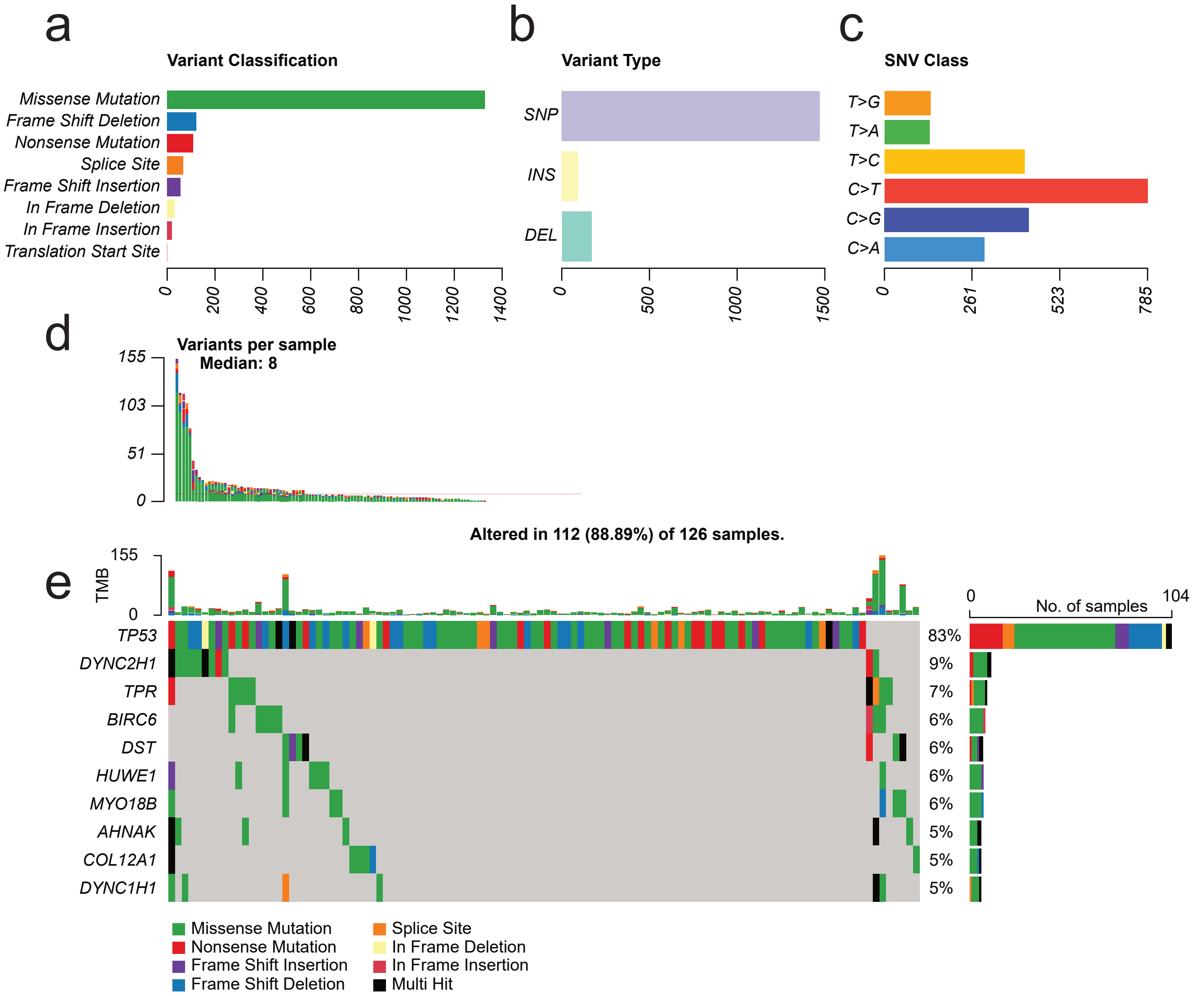

Oncoplot showing the top 10 most frequently mutated genes in TCGA triple-negative samples for genes in the MDA-MB231-exclusive PPI

Supplementary Figure 7: The mutational landscape of Triple-negative breast cancer PPIs as derived from the TCGA-database, showing in **a)** and **b)** the quantitative distribution of the specific variant types and broader classes among 112 out of 126 clinical triple-negative breast tumor cases. **c)** The type of single nucleotide variant (SNV) and associated frequencies are indicated. **d)** Bar-plot indicating the number of variants (median =8) sequenced for each tumor sample and includes the specific variant types (color-code same as in **a.**). **e)** Oncoplot showing the top 10 MDA-MB-231-associated PPI genes most frequently mutated in triple-negative breast cancer cases along with the various types of mutations as catalogued in TCGA. Source data are provided as a source data file.

Supplementary References

1. Havugimana PC*, et al.* A census of human soluble protein complexes. *Cell* **150**, 1068-1081 (2012).
2. Wan C*, et al.* Panorama of ancient metazoan macromolecular complexes. *Nature* **525**, 339-344 (2015).
3. Huttlin EL*, et al.* Dual proteome-scale networks reveal cell-specific remodeling of the human interactome. *Cell* **184**, 3022-3040 e3028 (2021).
4. Skinnider MA*, et al.* An atlas of protein-protein interactions across mouse tissues. *Cell* **184**, 4073-4089 e4017 (2021).
5. Drew K, Wallingford JB, Marcotte EM. hu.MAP 2.0: integration of over 15,000 proteomic experiments builds a global compendium of human multiprotein assemblies. *Mol Syst Biol* **17**, e10016 (2021).
