# Supplementary material for: Scalable multiplex co-fractionation/mass spectrometry platform for accelerated protein interactome discovery
Source: Nat Commun. 2022 Jul 13;13:4043. doi: 10.1038/s41467-022-31809-z (PMC9279285; doi:10.1038/s41467-022-31809-z)
Supplement: Supplementary file 2 — Description of Additional Supplementary Files [file 41467_2022_31809_MOESM2_ESM.docx]

File Name: Supplementary Data 1

Description: **LC-MS/MS proteins identification by multiplex CF/MS.** List of 4,613 total proteins identified globally across the 1,152 IEX-HPLC fractions from the three cell lines examined (192 fractions per line x 2 replicates x 3 lines).

File Name: Supplementary Data 2

Description: **EPIC-derived** **high confidence PPIs with physical and functional supporting evidence.** List of 25,235 EPIC-derived PPIs. The UniprotKB accession and gene names for all interacting proteins are indicated. The lowest default EPIC PPI score calculated by the random forest classifier within EPIC is 0.5. EPIC-scores (≥ 0.5) associated with PPIs are indicated for the respective cell lines. PPIs with a null score indicate that these could not meet the minimum threshold of 0.5 and are therefore considered as least likely to exist for the given cell line. “Y” denotes documentation/annotation of the protein interaction in the indicated public repositories or literature.

File Name: Supplementary Data 3

Description: **Predicted protein complexes, including new and annotated complexes.** Predicted complexes for the MCF10A, MCF7, and MDA-MB-231 cells are respectively shown in **Spreadsheets 1, 2, and 3.** **Spreadsheet 4** shows a binary pairwise comparison of protein complexes between the indicated cell line pairs. The maximum Simpson’s similarity score (Simpson’s index, SI) for each pairwise comparison is indicated. Protein complexes with SI ≥ 0.45 are considered overlapping and are indicated by Y or otherwise N (non-overlapping) in the table. **Spreadsheet 5** contains the list of putative complexes labelled as “Annotated” (SI > 0.45) or “Novel” (SI ≤ 0.45) based on match to curated databases (Corum, Reactome, GO CC, and IntAct) and previously published protein interactome studies by our group and others.

File Name: Supplementary Data 4

Description: **Functional gene set enrichment analysis of protein complexes.** List of enriched cellular pathways and processes (p-value < 0.05) associated with member proteins of differential protein complexes for each cell line. Leading edge proteins of each annotated pathway or functional term are also shown.

﻿
