# Supplementary material for: Scalable multiplex co-fractionation/mass spectrometry platform for accelerated protein interactome discovery
Source: Nat Commun. 2022 Jul 13;13:4043. doi: 10.1038/s41467-022-31809-z (PMC9279285; doi:10.1038/s41467-022-31809-z)
Supplement: Supplementary file 7 — Reporting Summary [file 41467_2022_31809_MOESM7_ESM.pdf]

nature portfolio

Corresponding author(s): Dr. Andrew Emili
Last updated by author(s): Jun 13, 2022

# Reporting Summary

Nature Portfolio wishes to improve the reproducibility of the work that we publish. This form provides structure for consistency and transparency in reporting. For further information on Nature Portfolio policies, see our Editorial Policies and the Editorial Policy Checklist.

## Statistics

For all statistical analyses, confirm that the following items are present in the figure legend, table legend, main text, or Methods section.

| n/a | Confirmed | |
|---|---|---|
| ☐ | ☒ | The exact sample size (*n*) for each experimental group/condition, given as a discrete number and unit of measurement |
| ☐ | ☒ | A statement on whether measurements were taken from distinct samples or whether the same sample was measured repeatedly |
| ☒ | ☐ | The statistical test(s) used AND whether they are one- or two-sided<br>*Only common tests should be described solely by name; describe more complex techniques in the Methods section.* |
| ☒ | ☐ | A description of all covariates tested |
| ☒ | ☐ | A description of any assumptions or corrections, such as tests of normality and adjustment for multiple comparisons |
| ☒ | ☐ | A full description of the statistical parameters including central tendency (e.g. means) or other basic estimates (e.g. regression coefficient) AND variation (e.g. standard deviation) or associated estimates of uncertainty (e.g. confidence intervals) |
| ☐ | ☒ | For null hypothesis testing, the test statistic (e.g. *F*, *t*, *r*) with confidence intervals, effect sizes, degrees of freedom and *P* value noted<br>*Give P values as exact values whenever suitable.* |
| ☒ | ☐ | For Bayesian analysis, information on the choice of priors and Markov chain Monte Carlo settings |
| ☒ | ☐ | For hierarchical and complex designs, identification of the appropriate level for tests and full reporting of outcomes |
| ☐ | ☒ | Estimates of effect sizes (e.g. Cohen's *d*, Pearson's *r*), indicating how they were calculated |

*Our web collection on statistics for biologists contains articles on many of the points above.*

## Software and code

Policy information about availability of computer code

| | |
|---|---|
| Data collection | We used the Q Exactive Orbitrap HF instrument (ThermoFisher Scientific) to collect all raw mass spectormetry data |
| Data analysis | We used MaxQuant (Version 1.6.1.0) to process and search raw mass spec data. MaxQuant is publicly available. EPIC software (no version number assigned) was used to infer PPI and protein complexes. EPIC is publicly available via the docker container (https://hub.docker.com/r/baderlab/bio-epic/). Modelling of the protein complexes was performed using publicly available servers, AlphaFold2 (https://alphafold.ebi.ac.uk/) and ClusProTBM (https://tbm.cluspro.org). Multiple sequence alignments of protein sequences were done using the MMseqs2 tool, Version 13-45111 (https://github.com/soedinglab/MMseqs2).<br>The R package "Maftools" was used to analyze the different mutations (single nucleotide polymorphisms, deletions and insertions) associated with PPI genes. The R code for Maftools is publicly available via Github (https://github.com/PoisonAlien/Maftools) and is installable via Bioconductor project (https://bioconductor.org/packages/release/bioc/html/maftools.html). |

For manuscripts utilizing custom algorithms or software that are central to the research but not yet described in published literature, software must be made available to editors and reviewers. We strongly encourage code deposition in a community repository (e.g. GitHub). See the Nature Portfolio guidelines for submitting code & software for further information.

## Data

Policy information about availability of data

All manuscripts must include a data availability statement. This statement should provide the following information, where applicable:

- Accession codes, unique identifiers, or web links for publicly available datasets
- A description of any restrictions on data availability
- For clinical datasets or third party data, please ensure that the statement adheres to our policy

The mass spectrometry proteomics data (including both raw MS data and processed MaxQuant output) have been deposited to the ProteomeXchange Consortium via the PRIDE partner repository with the dataset identifier PXD027704. Processed data are included as Supplementary Tables.
These data can be accessed via the weblink, https://www.ebi.ac.uk/pride/archive/projects/PXD027704/private, and using the following PRIDE reviewer login information:
Identifier: PXD027704
Username: reviewer_pxd027704@ebi.ac.uk
Password: WkKuIx8F

## Human research participants

Policy information about studies involving human research participants and Sex and Gender in Research.

| | |
|---|---|
| Reporting on sex and gender | Not applicable |
| Population characteristics | Not applicable |
| Recruitment | Not applicable |
| Ethics oversight | Not applicable |

Note that full information on the approval of the study protocol must also be provided in the manuscript.

# Field-specific reporting

Please select the one below that is the best fit for your research. If you are not sure, read the appropriate sections before making your selection.

[x] Life sciences [ ] Behavioural & social sciences [ ] Ecological, evolutionary & environmental sciences

For a reference copy of the document with all sections, see nature.com/documents/nr-reporting-summary-flat.pdf

# Life sciences study design

All studies must disclose on these points even when the disclosure is negative.

| | |
|---|---|
| Sample size | Three cell lines were used in the mCF-MS experiments. Two of these are breast cancer cell lines and one is a mammary gland-derived non-transformed/non-ongogenic cell line which was used as a control. For each cell line, two technical replicates were included and 192 biochemical fractions were collected for each replicate which represent sufficient sample size. |
| Data exclusions | All raw mass spec data were included in the analyses. |
| Replication | Two independent fractionations (i.e. replicate ion-exchange chromatography) were performed for each cell line as described in the manuscript. High correlation between the replicates was achieved as calculated by Pearson's correlation. |
| Randomization | There was no randomization in sample preparation, data acquisition or analyses. Fractions were multiplexed via TMT and injected sequentially in the MS. All acquired raw data were analyzed on MaxQuant. Randomization was not required as samples were uniquely barcoded with distinct TMT tags and then multiplexed together for subsequent analyses. |
| Blinding | We used supervised machine learning classifier to predict PPIs and protein complexes. Thus blinding is not relevant to this study. |

# Reporting for specific materials, systems and methods

We require information from authors about some types of materials, experimental systems and methods used in many studies. Here, indicate whether each material, system or method listed is relevant to your study. If you are not sure if a list item applies to your research, read the appropriate section before selecting a response.

## Materials & experimental systems

| n/a | Involved in the study |
| --- | --- |
| ☒ | ☐ Antibodies |
| ☐ | ☒ Eukaryotic cell lines |
| ☒ | ☐ Palaeontology and archaeology |
| ☒ | ☐ Animals and other organisms |
| ☒ | ☐ Clinical data |
| ☒ | ☐ Dual use research of concern |

## Methods

| n/a | Involved in the study |
| --- | --- |
| ☒ | ☐ ChIP-seq |
| ☒ | ☐ Flow cytometry |
| ☒ | ☐ MRI-based neuroimaging |

# Eukaryotic cell lines

Policy information about cell lines and Sex and Gender in Research

| | |
| --- | --- |
| Cell line source(s) | Authenticated MCF10A, MCF7 and MDA-MB-231 cell lines were obtained from ATCC. |
| Authentication | No further authentication was performed. |
| Mycoplasma contamination | Cell lines were not tested for mycoplasma. |
| Commonly misidentified lines (See ICLAC register) | There is no ICLAC report about the misidentification or cross-contamination of the above cell lines. |
